# Calcidiol Deficiency in End-Stage Organ Failure and after Solid Organ Transplantation: *Status quo*

**DOI:** 10.3390/nu5072352

**Published:** 2013-07-01

**Authors:** Ursula Thiem, Bartosz Olbramski, Kyra Borchhardt

**Affiliations:** Division of Nephrology and Dialysis, Department of Internal Medicine III, Medical University of Vienna, Spitalgasse 23, Vienna 1090, Austria; E-Mails: n0642244@students.meduniwien.ac.at (B.O.); kyra.borchhardt@meduniwien.ac.at (K.B.)

**Keywords:** vitamin D, heart failure, pulmonary disease, liver failure, chronic kidney disease, transplantation, calcidiol, rejection, supplementation, guidelines

## Abstract

Among patients with organ failure, vitamin D deficiency is extremely common and frequently does not resolve after transplantation. This review crystallizes and summarizes existing data on the *status quo* of vitamin D deficiency in patients with organ failure and in solid organ transplant recipients. Interventional studies evaluating different treatment strategies, as well as current clinical practice guidelines and recommendations on the management of low vitamin D status in these patients are also discussed.

## 1. Introduction

Vitamin D deficiency is a commonly observed phenomenon in patients with organ failure and solid organ transplant recipients. It occurs in patients with different types of solid organ transplant and frequently persists even in the long-term, post-transplant period (reviewed in [1]). The causes of vitamin D deficiency in these patients are diverse. Vitamin D deficiency may be primarily ascribed to lifestyle and environmental factors that result in reduced exposure to sunlight, as the main source of vitamin D is the skin, where it is synthesized from 7-dehydrochoesterol under the influence of ultraviolet light (reviewed in [2]). On the other hand, in patients with end-stage organ disease, there may be additional disease-specific factors that contribute to vitamin D deficiency, such as liver dysfunction [3] or uremia, which reduces the capacity of the skin to synthesize vitamin D [4,5]. After transplantation, the avoidance of sunlight, due to the increased risk of skin cancer in immunosuppressed patients [6], may be the main factor causing vitamin D deficiency [7]. Additional factors might involve the use of glucocorticoids, which were shown to enhance the catabolism of calcidiol [8].

Vitamin D deficiency is also commonly observed in the general population [9]. The National Health and Nutrition Examination Survey (NHANES) between 2002 and 2004, for example, assessed calcidiol levels in a representative sample of more than 20,000 persons in the USA and revealed that calcidiol levels below 20 ng/mL occur in approximately one third of the studied population [10]. In non-institutionalized elderly people across 11 European countries, 36% of men and 47% of women showed calcidiol levels below 12 ng/mL during winter [11]. For the general population, the Institute of Medicine in 2011 released their report on dietary reference intakes for calcium and vitamin D. For optimal bone health, the recommended dietary allowances of 600 International Units of vitamin D for ages up to 70 years and 800 International Units for ages above 70 years are suggested, corresponding to calcidiol levels above 20 ng/mL [12].

Herein, we review the prevalence of vitamin D deficiency in patients with end-stage organ failure and organ transplant recipients, as well as clinical trials on supplementation strategies and current guidelines on the recommendation of vitamin D intake in these patients. In this review, we consider 25-hydroxyvitamin D (calcidiol) levels below 30 ng/mL as insufficiency or hypovitaminosis, below 20 ng/mL as deficiency and below 10 ng/mL as severe deficiency (×2.5 for conversion to nmol/L).

## 2. Congestive Heart Failure and Cardiac Transplantation

### 2.1. Vitamin D Status in Patients with End-Stage Heart Failure

Hypovitaminosis D is highly prevalent among patients with congestive heart failure, with 17% to 57% of the patients displaying severe vitamin D deficiency [13,14]. The vitamin D status was reported to be related to the severity of the disease. In particular, patients evaluated for cardiac transplantation and classified United Network of Organ Sharing (UNOS) status 1 (*i.e.*, hospitalization and dependence on intravenous inotropic agents or left ventricular assist devices) had significantly lower calcidiol levels as compared with patients classified UNOS status 2, who were well enough to be managed as outpatients, (19 ng/mL *vs.* 24 ng/mL). While 23% of status 1 patients displayed severe vitamin D deficiency, only 8% of status 2 patients did so [13]. Similarly, mean serum calcidiol levels were reported to be lower in end-stage congestive heart failure patients awaiting cardiac transplantation who were classified as urgent or high urgent candidates according to the Eurotransplant listing criteria as compared with elective candidates (9.3 ng/mL *vs.* 14 ng/mL). None of the urgent or high urgent and only about 5% of the elective transplant candidates had sufficient vitamin D levels. Severe vitamin D deficiency was present in 57% of urgent or high urgent and in 50% of elective transplant candidates [14].

### 2.2. Vitamin D Status in Cardiac Transplant Recipients

Only one study reported the vitamin D status at the time of cardiac transplantation. Almost 90% of the patients presented with vitamin D insufficiency and 10% were found to be severely deficient [15]. In short-term heart transplant recipients, mean serum calcidiol levels increased from 18.7 ng/mL, analyzed within 12 months pre-transplant, to 24.5 ng/mL at one year post-transplant. Even though intake of 400 to 800 International Units of vitamin D was recommended to all patients, approximately three quarters of the patients displayed vitamin D insufficiency at one year post-transplant [16]. Similar results were obtained from an Iranian cohort of short-term heart transplant recipients, where two thirds were reported to be vitamin D deficient [17]. Even in the long-term post-transplant period, vitamin D deficiency frequently persists. In cardiac transplant recipients with a mean allograft age of approximately four years, more than 90% of the patients were reported to have hypovitaminosis D, with more than one third of patients displaying severe deficiency [18]. A summary of the vitamin D status in patients with end-stage heart failure and cardiac transplant recipients is presented in Table 1.

### 2.3. Interventional Studies and Guidelines

Despite the high prevalence of vitamin D insufficiency among patients with advanced heart failure and cardiac transplant recipients, interventional studies are sparse or lacking. In a randomized controlled trial in patients with heart failure (New York Heart Association class II and higher), the effect of daily 500 mg calcium and 2000 International Units vitamin D_3_ on survival, cytokine profiles and echocardiographic parameters was studied and compared with calcium treatment alone. After nine months, treatment with vitamin D_3_ increased the mean serum calcidiol level by 26.8 ng/mL, while an increase of only 3.6 ng/mL was observed in placebo treated patients. Treatment with vitamin D_3_ prevented an increase in tumor necrosis factor alpha, as observed in placebo-treated patients, and increased interleukin 10 levels [19]. Moreover, a recent study investigated the effect of vitamin D_3_ on biochemical and functional parameters of congestive heart failure in vitamin D insufficient patients with New York Heart Association class I to III heart failure. The treatment consisted of 50,000 International Units per week for eight weeks. Thereafter, the patients received 50,000 International Units every month for two months. Mean serum calcidiol level increased by 17 ng/mL after the four month treatment period. Interestingly, a decrease in pro-brain natriuretic peptide and high-sensitivity C-reactive protein, as well as an improvement in six minute walk distance and New York Heart Association class was observed [20].

Current guidelines for vitamin D intake in heart transplant candidates and cardiac transplant recipients are based on the beneficial effects of vitamin D therapy on corticosteroid-induced bone loss. In particular, based on expert consensus (Level of Evidence C), the International Society of Heart and Lung Transplantation recommends a daily intake of 1000 to 1500 mg of calcium and 400 to 1000 International Units of vitamin D to all heart transplant candidates and recipients. Serum calcidiol levels should be maintained above 30 ng/mL [21].

**Table 1:** Vitamin D status in congestive heart failure and cardiac transplant patients.

Ref.	Country, State	*N*	Study design and study population	Time point of calcidiol analysis	Mean ± SD calcidiol (ng/mL)	% insufficient (<30 ng/mL)	% deficient (<20 ng/mL)	% severely deficient (<10 ng/mL)	Supplementation
[13]	USA, New York	101	cross-sectional study; severe heart failure patients (NYHA III, IV) referred for tx		21			17	5% took supplemental calcium or vitamin D
[14]	Germany	383	cross-sectional study; heart failure patients awaiting tx (elective *vs*. urgent candidates)		14 *vs*.9.3	95.4 *vs*. 100	86.2 *vs*. 94.8	50.2 *vs*. 56.9	no
[15]	USA, New York	46	randomized controlled trial; heart transplant recipients	10 ± 7 days after tx	19.1 ± 8.3	89	64.5	9.5	800–1000 IU vitamin D once oral medication was tolerated
[16]	Norway	59	randomized controlled trial; heart transplant recipients	pre-transplant *vs.* 1 year after tx	18.7 *vs.* 24.6	73.6			400–800 IU vitamin D and 1000 mg calcium daily recommended
[17]	Iran	26	retrospective study; heart transplant recipients	24.8 ± 21 months after tx	17.8 ± 10.5		66.6		no
[18]	Italy	180	cross-sectional study; heart transplant recipients	3.91 years after tx	14.33 ± 8.25	92		35.5	no

tx, transplantation; NYHA, New York Heart Association; IU, International Units.

## 3. End-Stage Pulmonary Disease and Lung Transplantation

### 3.1. Vitamin D Status in Patients with End-Stage Pulmonary Disease

In end-stage pulmonary disease patients, varying prevalence of vitamin D deficiency was reported depending on the underlying disease. Severe vitamin D deficiency was seen in 14% to 40% of patients with cystic fibrosis [22,23,24], 20% to 42% of patients with chronic obstructive pulmonary disease [22,23], 14% of patients with pulmonary fibrosis [23] and 18% of patients with pulmonary hypertension [23]. Even though in one study, the majority of the patients received multivitamin supplements containing 400 to 800 International Units of vitamin D, 40% had severe vitamin D deficiency, and only 25% of the patients showed serum calcidiol levels above 20 ng/mL [24].

### 3.2. Vitamin D Status in Lung Transplant Recipients

In short-term lung transplant recipients in the United States, the proportion of patients with vitamin D insufficiency decreased from 79% at the time of transplantation to 26% at one year post-transplant. Approximately half of the patients received vitamin D supplements at the time of transplantation, while all of them did so after lung transplantation. Vitamin D deficiency at the time of transplantation was associated with an increased risk of experiencing acute rejection episodes or infections [25]. Similarly, a European study reported an improvement in vitamin D status after one year of lung transplantation. Mean serum calcidiol levels increased from 25.1 ng/mL at the time of transplantation to 29.4 ng/mL at one year post-transplant. Intake of 400 to 800 International Units of vitamin D was recommended to all patients. Still, approximately half of the patients displayed vitamin D insufficiency at one year post-transplant [16].

Data on the vitamin D status in long-term lung transplant recipients is sparse. In a Belgian cohort of 131 prevalent lung transplant recipients with an allograft age ranging from one to four years, approximately half of the patients were reported to have vitamin D insufficiency, despite daily treatment with 880 to 1000 International Units of cholecalciferol for the prevention of osteoporosis. A subgroup-analysis revealed that the proportion of vitamin D insufficient patients was similar between patients with one, two, three or four years of follow-up (ranging from 42% at one and two years post-transplant to 53% at three and four years post-transplant). Interestingly, after multivariate adjustment, vitamin D deficiency was associated with lower FEV_1_ (forced expiratory volume in one second), and patients deficient in vitamin D experienced more episodes of moderate to severe B-grade rejection (lymphocytic bronchiolitis) [26]. Details on the vitamin D status in patients with end-stage pulmonary disease and lung transplant recipients are summarized in Table 2.

**Table 2:** Vitamin D status in end-stage pulmonary disease and lung transplant patients.

Ref.	Country, State	N	Study design and study population	Time point of calcidiol analysis	Mean ± SD calcidiol (ng/mL)	% insufficient (<30 ng/mL)	% deficient (<20 ng/mL)	% severely deficient (<10 ng/mL)	Supplementation
[22]	USA, New York	70	cross-sectional study; lung transplant candidates (COPD/cystic fibrosis/other)		20/17/14			20/36/20	no
[23]	Switzerland	63	cross-sectional study; end-stage lung disease patients		18 ± 11			25	no
[24]	USA, New York	20	cross-sectional study; lung transplant candidates		16		75	40	80% received 400–800 IU vitamin D
[27]	Norway	71	cross-sectional study; lung transplant candidates (normal *vs.* underweight)		14.9 ± 6.3 *vs*. 15.2 ± 6.6	100 *vs.* 97	55 *vs.* 52 ^a^		no
[28]	USA, North Carolina	70	retrospective study; end-stage cystic fibrosis patients referred for tx		20.9 ± 11.9				90% received vitamin D in the form of ADEK
[25]	USA, Illinois	109	retrospective study; lung transplant recipients	near-transplant period *vs.* 1 year after tx	28.2	79.4 *vs.* 26.5			1000 IU daily up to 50,000 IU once or twice weekly in 50% before, 100% after tx
[16]	Norway	35	randomized controlled trial; lung transplant recipients	pre-transplant *vs.* 1 year after tx	25.1 *vs.* 29.4	50			400–800 IU vitamin D and 1000 mg calcium daily recommended
[26]	Belgium	131	cross-sectional study; lung transplant recipients	12–48 months after tx		47.3	19.8	4.6	880–1000 IU vitamin D

tx, transplantation; IU, International Units; ^a^ <12.5 ng/mL.

### 3.3. Interventional Studies and Guidelines

Based on the finding that vitamin D deficiency in lung transplant recipients is associated with an increased risk of developing rejection episodes, a randomized controlled trial is currently investigating the effect of a two year therapy of monthly 100,000 International Units of vitamin D in incident lung transplant recipients on the development of bronchiolitis obliterans syndrome, a frequent manifestation of chronic rejection (clinicaltrials.gov NCT01212406).

To our knowledge, there are no guidelines on the management of vitamin D deficiency in patients with end-stage pulmonary disease or lung transplant patients.

## 4. Liver Failure and Liver Transplantation

### 4.1. Vitamin D Status in Patients with End-Stage Liver Disease

The liver is the main site where hydroxylation of vitamin D at position C-25 takes place. Thus, it is not surprising that the degree of liver dysfunction correlates with calcidiol levels [3] and that the prevalence of vitamin D insufficiency is particularly high in patients with chronic liver disease [29,30,31,32]. At the time of transplantation, between 80% and 95% of the patients with end-stage liver failure were reported to have hypovitaminosis D, with varying prevalence of severe vitamin D deficiency (ranging from 3% up to 50%) [15,33,34,35]. Notably, in one study, more than one fifth of the patients had serum calcidiol below the detection limit of 6.8 ng/mL [15].

### 4.2. Vitamin D Status in Liver Transplant Recipients

At three months after liver transplantation, Reese and colleagues observed a marked increase in serum calcidiol levels with a median change of 17.8 ng/mL (interquartile range 8.6 to 25.9 ng/mL). The prevalence of vitamin D deficiency dropped from 84% at the time of transplantation to 24% after three months post-transplant. Moreover, serum vitamin D binding protein and albumin substantially increased, which according to the authors, might have contributed to the marked improvement in vitamin D status by facilitating a shift of calcidiol from the adipose tissue to the circulation. Even though the prevalence of vitamin D deficiency was similar in black and non-black patients at the time of transplantation, median calcidiol levels were significantly lower in black patients (4.9 *vs.* 9.6 ng/mL). At three months after transplantation, vitamin D deficiency was more prevalent among black liver transplant recipients as compared with non-black patients (38% *vs.* 20%) [36]. In contrast, in an Iranian cohort of liver transplant recipients, a high prevalence of vitamin D deficiency persisted in the early post-transplant period [17]. In a Spanish study, 45 liver transplant recipients were followed up to three years after transplantation. Before transplantation, severe vitamin D deficiency was present in 62% of the patients, with a mean serum calcidiol of 9.4 ng/mL. In comparison, 40 healthy age-matched controls displayed a mean serum calcidiol of 23.1 ng/mL. Serum calcidiol levels continuously increased over time (9.5 ng/mL at one month, 16.5 ng at three months, 15.9 ng at six months, 19 ng/mL at 12 months, 19.9 ng/mL at 18 months, 18 ng/mL at 24 months and 19.5 ng/mL at 36 months post-transplant). At one year and three years after transplantation, severe vitamin D deficiency was observed in only 14% and 10% of the patients, respectively [37]. In contrast, in an Israelian cohort of long-term liver transplant recipients with a mean allograft age of 7.5 years, more than one third was reported to have severe vitamin D deficiency [38]. Table 3 summarizes the prevalence of vitamin D insufficiency, deficiency and severe deficiency in patients with end-stage liver disease and liver transplant recipients.

### 4.3. Interventional Studies and Guidelines

Recently, a clinical practice guideline on the evaluation, treatment and prevention of vitamin D deficiency has been published by the Endocrine Society. According to this guideline, patients with hepatic failure are considered at high risk for vitamin D deficiency. Therefore, calcidiol measurements are reasonable, and supplementation in case of calcidiol levels below 20 ng/mL is recommended. For bone health, adults are considered to require at least 600 to 800 International Units daily. For reaching calcidiol levels above 30 ng/mL, however, higher doses may be required (1500 to 2000 International Units). This recommendation is based on lower quality evidence [39]. For the management of liver transplant patients, the American Association for the Study of Liver Disease and the American Society of Transplantation released a practice guideline, which recommends calcidiol levels to be maintained above 30 ng/mL for optimal bone health. This daily use of 400 to 1000 International Units of vitamin D is suggested; however, calcidiol levels should be measured at least annually to check if the treatment regimen is appropriate. Treatment with vitamin D supplements is recommended in osteopenic liver transplant recipients with a Level of Evidence A [40].

## 5. Chronic Kidney Disease and Kidney Transplantation

### 5.1. Vitamin D Status in Patients with Chronic Kidney Disease

Vitamin D deficiency is very common in patients with chronic kidney disease (CKD) across all stages. In North America, a prevalence of vitamin D insufficiency of approximately 85% was reported among patients with advanced kidney disease [41,42]. Severe vitamin D deficiency (<15 ng/mL) was more pronounced in patients with CKD stage 5 (56%), as compared with CKD stage 4 (37%) [42], and mean serum calcidiol was reported to be significantly lower in diabetic as compared with non-diabetic patients [43]. Similarly, in a cohort of chronic kidney disease stage 3 and 4 in the UK, hypovitaminosis D was very common, with 80% of the patients showing vitamin D insufficiency and 37% severe vitamin D deficiency (<15 ng/mL). After exclusion of patients who received vitamin D supplements or other drugs known to interfere with calcidiol levels, mean serum calcidiol remained unchanged. Likewise, the proportional distribution of patients with vitamin D insufficiency and severe deficiency was similar [44].

**Table 3:** Vitamin D status in end-stage liver disease and liver transplant patients.

Ref.	Country, State	*N*	Study design and study population	Time point of calcidiol analysis	Mean ± SD calcidiol (ng/mL)	% insufficient (<30 ng/mL)	% deficient (<20 ng/mL)	% severely deficient (<10 ng/mL)	Supplementation
[15]	USA, New York	23	randomized controlled trial; liver transplant recipients	10 ± 7 days after tx	13.8 ± 7	95	83	30	800–1000 IU vita­min D once oral medication was tolerated
[17]	Iran	23	retrospective study; liver transplant recipients	21.6 ± 21.4 months after tx	14.3 ± 9.6		80.9		no
[33]	Italy	93	retrospective study; liver transplant recipients	at the time of tx	12.5 (1.0–48.5) ^a^	92.5		49.5 ^d^	60% took 800 IU vitamin D, 40% started in the first month after tx
[34]	USA, Wisconsin	63	retrospective study; liver disease patients awaiting tx			81 ^b^	75	6.3	10% took vitamin D supplements
[35]	Australia	107	prospective cohort study; liver disease patients assessed for tx (cholestatic *vs.* hepatocellular disease)		46.5 ^a^ *vs.* 42		66	15	5% took vitamin D supplements
[36]	USA, Pennsylvania	202	prospective cohort study; liver transplant recipients	at the time of tx *vs.* 3 months after tx	6.7 *vs.* 27.4 ^a^		84 *vs.* 24		all received 400 IU vitamin D
[37]	Spain	45	prospective study; liver transplant candidates	at the time of tx	9.4			62	no
		39		1 year after tx	19			14	
		34		2 years after tx	18				
		30		3 years after tx	19.5			10	
[38]	Israel	29	cross-sectional study; liver transplant patients	7.5 ± 4.1 years after tx			65.5 ^c^	38	0.25 µg alphacalcidol pre-transplant

tx, transplantation; IU, International Units; ^a^ median (range), ^b^ <32 ng/mL; ^c^ <15 ng/mL; ^d^ <12.5 ng/mL.

### 5.2. Vitamin D Status in Kidney Transplant Recipients

Only a few studies investigated the vitamin D status at the time of transplantation. Almost 90% of incident kidney transplant recipients were reported to have insufficient calcidiol levels, with a mean serum calcidiol of 16.6 ng/mL and a trend towards higher calcidiol levels in summer as compared with winter. Notably, vitamin D levels of black kidney transplant recipients were significantly lower compared with non-black patients (13.6 ng/mL *vs*. 17.5 ng/mL) [45]. Similar findings were obtained from another cohort of African American renal transplant recipients in the early post-transplant period, where 95% of the patients presented with calcidiol levels below 30 ng/mL and 58% showed severe vitamin deficiency (<16 ng/mL), even at the end of summer [46].

Even though vitamin D status was reported to improve in the early post-transplant period [47], low vitamin D levels are frequently observed in long-term kidney transplant recipients [7,48,49]. Querings *et al*. investigated the vitamin D status of 31 long-term kidney transplant recipients with a mean allograft age of seven years at the end of winter and found a mean serum calcidiol of 10.9 ng/mL. Notably, vitamin D sufficiency was observed in one patient only, and almost one third of the patients had serum calcidiol levels below the detection limit (<4 ng/mL) [7]. In contrast, in a Danish cohort of long-term kidney transplant recipients, approximately one fifth of the patients were found to have sufficient vitamin D during the winter months, with a median calcidiol level of 19.8 ng/mL. A subgroup analysis revealed that 60% of these patients received vitamin D supplements at a median dose of 7.8 µg per day (in the form of ergocalciferol or cholecalciferol or alphacalcidol), which might explain the lower prevalence of vitamin D insufficiency in this cohort. In addition, a median alimentary intake of approximately 3.2 µg vitamin D per day was reported [49]. Moreover, clear seasonal variations in serum calcidiol levels were found in long-term kidney transplant recipients, with 3.5-times more patients reaching calcidiol above 30 ng/mL during summer months compared with winter months. Still, the majority of the patients exhibited vitamin D insufficiency, even during summer [50,51]. In Table 4, details on the vitamin D status in CKD patients and renal transplant patients are presented. 

**Table 4:** Vitamin D status in chronic kidney disease and renal transplant patients.

Ref.	Country	*N*	Study design and Study population	Time point of calcidiol analysis	Mean ± SD calcidiol (ng/mL)	% insufficient (<30 ng/mL)	%deficient (<20 ng/mL)	% severely deficient (<10 ng/mL)	Supplementation
[41]	USA, diverse regions	178	cross-sectional study; patients with CKD 3 *vs*. 4		23.3 ± 14.5 *vs.* 18.6 ± 13.3	71 *vs.* 83		14 *vs.* 26	no
[42]	Canada	168	prospective study; CKD patients		18.1 ^a^		34.5 ^b^		active vitamin D analogs
[43]	Japan	76	prospective study; non-dialyzed CKD patients (non-diabetic *vs.* diabetic)		22.3 ± 9.4 *vs.* 11.4 ± 5.6				no
[44]	UK	112	cross-sectional study; patients with CKD 3 and 4		20.8 ± 12	80	36 ^b^	6 ^c^	
[45]	USA, Massachusetts	112	prospective; renal transplant recipients	at the time of transplantation	16.6 ± 9.6	87.5		28.6	active vitamin D analogs
[46]	USA, Virginia	38	cross-sectional study; African American renal transplant recipients	23 ± 20 months after tx	16 ± 7.4	94.7	57.8 ^b^		no
[47]	Turkey	161	longitudinal cohort study; renal transplant recipients	pre-transplant *vs.* 6 months after tx	18.1 ± 4 *vs*. 22 ± 4.5	65.8			no
[48]	UK	104	cross-sectional study; renal transplant recipients	3.4 (1.9–12) months after tx	13.2 ± 7.6	97	68 ^b^	12 ^c^	no
		140		6 (1–24) years after tx	16.8 ± 8	94	51 ^b^	5 ^c^	no
[7]	Germany	31	cross-sectional study; renal transplant recipients *vs.* age/gender matched controls	7 (0.5–19) years after tx	10.9 *vs.* 20	96.8 *vs.* 80.6	80.6 *vs.* 54.8	35.5 *vs.* 12.9	no
[49]	Denmark	173	cross-sectional study; renal transplant recipients (female *vs.* male)	7.4 (3.3–12.7) years after tx	21.6 *vs*. 18.2 ^a^	74 *vs.* 88	26 *vs*. 33 ^b^	3 *vs*. 9 ^c^	69% of women and 51% of men took a median daily dose of 400 and 200 IU vitamin D, respectively
[50]	UK	266	cross-sectional study; renal transplant recipients (winter *vs.* summer)	16 (12–23) years after tx	15.6 ± 10.8 *vs.* 23.6 ± 12.4	91 *vs.* 68	59 *vs.* 31 ^b^		10%–20% took alphacalcidol
[51]	Switzerland	50	prospective study; renal transplant recipients (winter *vs*. summer)	11.1 (0.8–33.6) years after tx	12.4 *vs.* 17.6 ^a^	96 *vs.* 86	90 *vs.* 60		61% of women and 51% of men took vitamin D

CKD, chronic kidney disease; tx, transplantation; IU, International Units; ^a^ median (range); ^b^ <16 ng/mL; ^c^ <5 ng/mL.

### 5.3. Interventional Studies and Guidelines

Even though vitamin D deficiency is extremely common among patients with chronic kidney disease, including renal transplant recipients, there is no consensus on how to treat vitamin D deficiency in these patients. Current guidelines of the National Kidney Foundation for patients with chronic kidney disease recommend the measurement of calcidiol in patients with CKD stage 3 or 4 only in case of elevated parathyroid hormone levels. Oral supplementation with ergocalciferol for six months, with 50,000 International Units per week for four weeks in the case of mild (5 to 15 ng/mL) and 12 weeks in the case of severe (below 5 ng/mL) vitamin D deficiency is proposed. Thereafter, a monthly dose of 50,000 International Units is recommended. For vitamin D insufficiency, ergocalciferol at a monthly dose of 50,000 International Units orally for six months is suggested. All of these recommendations are opinion-based [52]. For patients after renal transplantation, Clinical Practice Guidelines for the Care of Kidney transplant recipients (KDIGO) suggest correction of vitamin D deficiency or insufficiency as done for the general population (Level of Evidence C) [53].

A recent meta-analysis of seventeen observational and five randomized controlled trials in patients with various forms of CKD evaluated the effect of vitamin D supplementation and found a significant improvement of the vitamin D status, with a mean difference of 24.1 ng/mL in observational and 14 ng/mL in randomized controlled trials. Treatment regimens ranged from 4000 International Units daily up to 50,000 International Units daily, with an average treatment period of half a year [41].

The few studies available indicate that renal transplant recipients have a higher need for vitamin D to correct insufficiency than what is known from the general population. In particular, in a randomized controlled trial in kidney transplant recipients, Wissing and colleagues evaluated the effect of 400 mg/day calcium and 25,000 International Units of cholecalciferol per month on bone mineral density one year after transplantation. Surprisingly, this dose was not sufficient to correct vitamin D deficiency in these patients [54]. In contrast, Courbebaisse and colleagues used 100,000 International Units of cholecalciferol once every two weeks for two months and, thereafter, treated the patients with 100,000 International Units of cholecalciferol every two months for another six months. After the initial intensive treatment period, more than 90% of the patients showed calcidiol levels above 30 ng/mL, while only 50% were vitamin D sufficient after the maintenance treatment period, indicating that 100,000 International Units of cholecalciferol every two months is still not sufficient to maintain optimal vitamin D levels [55]. Moreover, these studies demonstrated that spontaneous recovery of vitamin D deficiency after kidney transplantation does not occur. In the untreated control groups, calcidiol either remained stable [55], or even decreased further [54], over time. Based on their previous study, the group of Courbebaisse established a pharmacokinetic model that describes calcidiol levels after treatment with cholecalciferol in kidney transplant recipients within the first year after transplantation. According to this model, a treatment regimen with six doses of 100,000 International Units cholecalciferol every two weeks, followed by 100,000 International Units once per month is proposed to maintain calcidiol levels between 30 and 80 ng/mL [56]. Several clinical trials are currently ongoing with different treatment regimens ([57], NCT00752401, NCT00748618, NCT01431430), which will help to identify strategies to maintain optimal vitamin D status.

## 6. Conclusions

In summary, studies in patients with organ failure consistently show that vitamin D insufficiency or deficiency is widespread among these patients, even in the healthier sub-population of patients awaiting transplantation. After transplantation, calcidiol levels frequently remain low and, in many cases, do not recover in the long-term post-transplant period. Minor discrepancies in the reported prevalence of vitamin D insufficiency or deficiency might result from the different treatment regimens used (if patients received vitamin D supplements or not), different habits and customs of the studied populations (sun protection and nutrition), but also from the different assays used to measure calcidiol.

From the current evidence, it is not clear whether vitamin D deficiency is one of the causative factors or a consequence in the development and progression of organ failure. For example, the active vitamin D metabolite, 1,25-dihydroxyvitamin D, might affect the development and progression of cardiovascular disease by different mechanisms of action, such as regulation of the mineral metabolism, interaction with the renin-angiotensin-aldosterone system or modulation of immune responses (reviewed in [58,59]). Similarly, it might exert renoprotective effects and, thus, delay the progression of CKD, e.g., by inhibition of the renin-angiotensin-aldosterone system, regulation of the immune system or increase of insulin sensitivity (reviewed in [60]). On the other hand, low calcidiol levels might simply reflect poorer health status in patients with advanced stages of organ failure.

At present, there are only a few recommendations on how to manage vitamin D deficiency in these patients; most of them are based on expert consensus and derived from the beneficial effects of vitamin D on the skeleton. However, especially in the setting of organ transplantation, the effects of vitamin D might go beyond bone health. In particular, the active metabolite, 1,25-dihydroxyvitamin D, has immunomodulatory activity, which is supported by extensive experimental research (reviewed in [61,62]). Recent clinical trials indicate that modulation of the immune system can be achieved by administration of nutritional vitamin D [63,64,65,66,67,68,69]. These immunomodulatory effects may be exploited in various diseases, including organ tolerance after transplantation. The increase in regulatory T-cells by nutritional vitamin D, as shown in a randomized controlled trial, could be of particular relevance in organ transplant recipients [63]. To date, however, we do not have sufficient evidence to recommend treatment with vitamin D based on its immunomodulatory actions, and further clinical trials are clearly warranted.

## References

[1] Stein E.M., Shane E. (2011). Vitamin D in organ transplantation. Osteoporos. Int..

[2] Holick M.F. (2007). Vitamin D deficiency. N. Engl. J. Med..

[3] Putz-Bankuti C., Pilz S., Stojakovic T., Scharnagl H., Pieber T.R., Trauner M., Obermayer-Pietsch B., Stauber R.E. (2012). Association of 25-hydroxyvitamin D levels with liver dysfunction and mortality in chronic liver disease. Liver Int..

[4] Nessim S.J., Jassal S.V., Fung S.V., Chan C.T. (2007). Conversion from conventional to nocturnal hemodialysis improves vitamin D levels. Kidney Int..

[5] Jacob A.I., Sallman A., Santiz Z., Hollis B.W. (1984). Defective photoproduction of cholecalciferol in normal and uremic humans. J. Nutr..

[6] Euvrard S., Kanitakis J., Claudy A. (2003). Skin cancers after organ transplantation. N. Engl. J. Med..

[7] Querings K., Girndt M., Geisel J., Georg T., Tilgen W., Reichrath J. (2006). 25-hydroxyvitamin D deficiency in renal transplant recipients. J. Clin. Endocrinol. Metab..

[8] Pascussi J.M., Robert A., Nguyen M., Walrant-Debray O., Garabedian M., Martin P., Pineau T., Saric J., Navarro F., Maurel P. (2005). Possible involvement of pregnane X receptor-enhanced CYP24 expression in drug-induced osteomalacia. J. Clin. Investig..

[9] Mithal A., Wahl D.A., Bonjour J.P., Burckhardt P., Dawson-Hughes B., Eisman J.A., El-Hajj Fuleihan G., Josse R.G., Lips P., Morales-Torres J. (2009). Global vitamin D status and determinants of hypovitaminosis D. Osteoporos. Int..

[10] Yetley E.A. (2008). Assessing the vitamin D status of the US population. Am. J. Clin. Nutr..

[11] Van der Wielen R.P., Lowik M.R., van den Berg H., de Groot L.C., Haller J., Moreiras O., van Staveren W.A. (1995). Serum vitamin D concentrations among elderly people in Europe. Lancet.

[12] Ross A.C., Manson J.E., Abrams S.A., Aloia J.F., Brannon P.M., Clinton S.K., Durazo-Arvizu R.A., Gallagher J.C., Gallo R.L., Jones G. (2011). The 2011 report on dietary reference intakes for calcium and vitamin D from the Institute of Medicine: What clinicians need to know. J. Clin. Endocrinol. Metab..

[13] Shane E., Mancini D., Aaronson K., Silverberg S.J., Seibel M.J., Addesso V., McMahon D.J. (1997). Bone mass, vitamin D deficiency, and hyperparathyroidism in congestive heart failure. Am. J. Med..

[14] Zittermann A., Schleithoff S.S., Gotting C., Dronow O., Fuchs U., Kuhn J., Kleesiek K., Tenderich G., Koerfer R. (2008). Poor outcome in end-stage heart failure patients with low circulating calcitriol levels. Eur. J. Heart Fail..

[15] Stein E.M., Cohen A., Freeby M., Rogers H., Kokolus S., Scott V., Mancini D., Restaino S., Brown R., McMahon D.J. (2009). Severe vitamin D deficiency among heart and liver transplant recipients. Clin. Transplant..

[16] Forli L., Bollerslev J., Simonsen S., Isaksen G.A., Kvamsdal K.E., Godang K., Gadeholt G., Pripp A.H., Bjortuft O. (2010). Dietary vitamin K2 supplement improves bone status after lung and heart transplantation. Transplantation.

[17] Movassaghi S., Nasiri Toosi M., Bakhshandeh A., Niksolat F., Khazaeipour Z., Tajik A. (2012). Frequency of musculoskeletal complications among the patients receiving solid organ transplantation in a tertiary health-care center. Rheumatol. Int..

[18] Dalle Carbonare L., Zanatta M., Braga V., Sella S., Vilei M.T., Feltrin G., Gambino A., Pepe I., Rossini M., Adami S. (2011). Densitometric threshold and vertebral fractures in heart transplant patients. Transplantation.

[19] Schleithoff S.S., Zittermann A., Tenderich G., Berthold H.K., Stehle P., Koerfer R. (2006). Vitamin D supplementation improves cytokine profiles in patients with congestive heart failure: A double-blind, randomized, placebo-controlled trial. Am. J. Clin. Nutr..

[20] Amin A., Minaee S., Chitsazan M., Naderi N., Taghavi S., Ardeshiri M. (2013). Can Vitamin D supplementation improve the severity of congestive heart failure?. Congest. Heart Fail..

[21] Costanzo M.R., Dipchand A., Starling R., Anderson A., Chan M., Desai S., Fedson S., Fisher P., Gonzales-Stawinski G., Martinelli L. (2010). The International Society of Heart and Lung Transplantation Guidelines for the care of heart transplant recipients. J. Heart Lung Transplant..

[22] Shane E., Silverberg S.J., Donovan D., Papadopoulos A., Staron R.B., Addesso V., Jorgesen B., McGregor C., Schulman L. (1996). Osteoporosis in lung transplantation candidates with end-stage pulmonary disease. Am. J. Med..

[23] Tschopp O., Boehler A., Speich R., Weder W., Seifert B., Russi E.W., Schmid C. (2002). Osteoporosis before lung transplantation: Association with low body mass index, but not with underlying disease. Am. J. Transplant..

[24] Donovan D.S., Papadopoulos A., Staron R.B., Addesso V., Schulman L., McGregor C., Cosman F., Lindsay R.L., Shane E. (1998). Bone mass and vitamin D deficiency in adults with advanced cystic fibrosis lung disease. Am. J. Respir. Crit. Care Med..

[25] Lowery E.M., Bemiss B., Cascino T., Durazo-Arvizu R.A., Forsythe S.M., Alex C., Laghi F., Love R.B., Camacho P. (2012). Low vitamin D levels are associated with increased rejection and infections after lung transplantation. J. Heart Lung Transplant..

[26] Verleden S.E., Vos R., Geenens R., Ruttens D., Vaneylen A., Dupont L.J., Verleden G.M., van Raemdonck D.E., Vanaudenaerde B.M. (2012). Vitamin D deficiency in lung transplant patients: Is it important?. Transplantation.

[27] Forli L., Bjortuft O., Boe J. (2009). Vitamin D status in relation to nutritional depletion and muscle function in patients with advanced pulmonary disease. Exp. Lung Res..

[28] Aris R.M., Renner J.B., Winders A.D., Buell H.E., Riggs D.B., Lester G.E., Ontjes D.A. (1998). Increased rate of fractures and severe kyphosis: Sequelae of living into adulthood with cystic fibrosis. Ann. Intern. Med..

[29] Arteh J., Narra S., Nair S. (2010). Prevalence of vitamin D deficiency in chronic liver disease. Dig. Dis. Sci..

[30] Malham M., Jorgensen S.P., Ott P., Agnholt J., Vilstrup H., Borre M., Dahlerup J.F. (2011). Vitamin D deficiency in cirrhosis relates to liver dysfunction rather than aetiology. World J. Gastroenterol..

[31] Miroliaee A., Nasiri-Toosi M., Khalilzadeh O., Esteghamati A., Abdollahi A., Mazloumi M. (2010). Disturbances of parathyroid hormone-vitamin D axis in non-cholestatic chronic liver disease: A cross-sectional study. Hepatol. Int..

[32] Fisher L., Fisher A. (2007). Vitamin D and parathyroid hormone in outpatients with noncholestatic chronic liver disease. Clin. Gastroenterol. Hepatol..

[33] Bitetto D., Fabris C., Falleti E., Fornasiere E., Fumolo E., Fontanini E., Cussigh A., Occhino G., Baccarani U., Pirisi M. (2010). Vitamin D and the risk of acute allograft rejection following human liver transplantation. Liver Int..

[34] Venu M., Martin E., Saeian K., Gawrieh S. (2013). High prevalence of vitamin A deficiency and vitamin D deficiency in patients evaluated for liver transplantation. Liver Transpl..

[35] Abbott-Johnson W.J., Kerlin P., Abiad G., Clague A.E., Cuneo R.C. (2011). Dark adaptation in vitamin A-deficient adults awaiting liver transplantation: Improvement with intramuscular vitamin A treatment. Br. J. Ophthalmol..

[36] Reese P.P., Bloom R.D., Feldman H.I., Huverserian A., Thomasson A., Shults J., Hamano T., Goral S., Shaked A., Olthoff K. (2012). Changes in vitamin D binding protein and vitamin D concentrations associated with liver transplantation. Liver Int..

[37] Monegal A., Navasa M., Guanabens N., Peris P., Pons F., Martinez de Osaba M.J., Ordi J., Rimola A., Rodes J., Munoz-Gomez J. (2001). Bone disease after liver transplantation: A long-term prospective study of bone mass changes, hormonal status and histomorphometric characteristics. Osteoporos. Int..

[38] Segal E., Baruch Y., Kramsky R., Raz B., Tamir A., Ish-Shalom S. (2003). Predominant factors associated with bone loss in liver transplant patients—After prolonged post-transplantation period. Clin. Transplant..

[39] Holick M.F., Binkley N.C., Bischoff-Ferrari H.A., Gordon C.M., Hanley D.A., Heaney R.P., Murad M.H., Weaver C.M. (2011). Evaluation, treatment, and prevention of vitamin D deficiency: An Endocrine Society clinical practice guideline. J. Clin. Endocrinol. Metab..

[40] Lucey M.R., Terrault N., Ojo L., Hay J.E., Neuberger J., Blumberg E., Teperman L.W. (2013). Long-term management of the successful adult liver transplant: 2012 practice guideline by the American Association for the Study of Liver Diseases and the American Society of Transplantation. Liver Transpl..

[41] LaClair R.E., Hellman R.N., Karp S.L., Kraus M., Ofner S., Li Q., Graves K.L., Moe S.M. (2005). Prevalence of calcidiol deficiency in CKD: A cross-sectional study across latitudes in the United States. Am. J. Kidney Dis..

[42] Ravani P., Malberti F., Tripepi G., Pecchini P., Cutrupi S., Pizzini P., Mallamaci F., Zoccali C. (2009). Vitamin D levels and patient outcome in chronic kidney disease. Kidney Int..

[43] Ishimura E., Nishizawa Y., Inaba M., Matsumoto N., Emoto M., Kawagishi T., Shoji S., Okuno S., Kim M., Miki T. (1999). Serum levels of 1,25-dihydroxyvitamin D, 24,25-dihydroxyvitamin D, and 25-hydroxyvitamin D in nondialyzed patients with chronic renal failure. Kidney Int..

[44] Stavroulopoulos A., Porter C.J., Roe S.D., Hosking D.J., Cassidy M.J. (2008). Relationship between vitamin D status, parathyroid hormone levels and bone mineral density in patients with chronic kidney disease stages 3 and 4. Nephrology (Carlton).

[45] Sadlier D.M., Magee C.C. (2007). Prevalence of 25(OH) vitamin D (calcidiol) deficiency at time of renal transplantation: A prospective study. Clin. Transplant..

[46] Tripathi S.S., Gibney E.M., Gehr T.W., King A.L., Beckman M.J. (2008). High prevalence of vitamin D deficiency in African American kidney transplant recipients. Transplantation.

[47] Yilmaz M.I., Sonmez A., Saglam M., Yaman H., Kilic S., Turker T., Unal H.U., Gok M., Cetinkaya H., Eyileten T. (2013). Longitudinal analysis of vascular function and biomarkers of metabolic bone disorders before and after renal transplantation. Am. J. Nephrol..

[48] Stavroulopoulos A., Cassidy M.J., Porter C.J., Hosking D.J., Roe S.D. (2007). Vitamin D status in renal transplant recipients. Am. J. Transplant..

[49] Ewers B., Gasbjerg A., Moelgaard C., Frederiksen A.M., Marckmann P. (2008). Vitamin D status in kidney transplant patients: Need for intensified routine supplementation. Am. J. Clin. Nutr..

[50] Penny H., Frame S., Dickinson F., Garrett G., Young A.R., Sarkany R., Chitalia N., Hampson G., Goldsmith D. (2012). Determinants of vitamin D status in long-term renal transplant patients. Clin. Transplant..

[51] Burkhalter F., Schaub S., Dickenmann M. (2012). Preserved circannual rhythm of vitamin D in kidney transplant patients. Swiss Med. Wkly..

[52] National Kidney Foundation (2003). K/DOQI clinical practice guidelines for bone metabolism and disease in chronic kidney disease. Am. J. Kidney Dis..

[53] KDIGO (2009). KDIGO clinical practice guideline for the care of kidney transplant recipients. Am. J. Transplant..

[54] Wissing K.M., Broeders N., Moreno-Reyes R., Gervy C., Stallenberg B., Abramowicz D. (2005). A controlled study of vitamin D3 to prevent bone loss in renal-transplant patients receiving low doses of steroids. Transplantation.

[55] Courbebaisse M., Thervet E., Souberbielle J.C., Zuber J., Eladari D., Martinez F., Mamzer-Bruneel M.F., Urena P., Legendre C., Friedlander G. (2009). Effects of vitamin D supplementation on the calcium-phosphate balance in renal transplant patients. Kidney Int..

[56] Benaboud S., Urien S., Thervet E., Prie D., Legendre C., Souberbielle J.C., Hirt D., Friedlander G., Treluyer J.M., Courbebaisse M. (2013). Determination of optimal cholecalciferol treatment in renal transplant recipients using a population pharmacokinetic approach. Eur J. Clin. Pharmacol..

[57] Thiem U., Heinze G., Segel R., Perkmann T., Kainberger F., Muhlbacher F., Horl W., Borchhardt K. (2009). VITA-D: Cholecalciferol substitution in vitamin D deficient kidney transplant recipients: A randomized, placebo-controlled study to evaluate the post-transplant outcome. Trials.

[58] Meems L.M., van der Harst P., van Gilst W.H., de Boer R.A. (2011). Vitamin D biology in heart failure: Molecular mechanisms and systematic review. Curr. Drug Targets.

[59] Pilz S., Tomaschitz A., Marz W., Drechsler C., Ritz E., Zittermann A., Cavalier E., Pieber T.R., Lappe J.M., Grant W.B. (2011). Vitamin D, cardiovascular disease and mortality. Clin. Endocrinol. (Oxf.).

[60] Shroff R., Wan M., Rees L. (2012). Can vitamin D slow down the progression of chronic kidney disease?. Pediatr. Nephrol..

[61] Di Rosa M., Malaguarnera M., Nicoletti F., Malaguarnera L. (2011). Vitamin D3: A helpful immuno-modulator. Immunology.

[62] Thiem U., Borchhardt K. (2011). Vitamin D in solid organ transplantation with special emphasis on kidney transplantation. Vitam. Horm..

[63] Bock G., Prietl B., Mader J.K., Holler E., Wolf M., Pilz S., Graninger W.B., Obermayer-Pietsch B.M., Pieber T.R. (2011). The effect of vitamin D supplementation on peripheral regulatory T cells and beta cell function in healthy humans: A randomized controlled trial. Diabetes Metab. Res. Rev..

[64] Hopkins M.H., Owen J., Ahearn T., Fedirko V., Flanders W.D., Jones D.P., Bostick R.M. (2011). Effects of supplemental vitamin D and calcium on biomarkers of inflammation in colorectal adenoma patients: A randomized, controlled clinical trial. Cancer Prev Res. (Phila.).

[65] Bucharles S., Barberato S.H., Stinghen A.E., Gruber B., Piekala L., Dambiski A.C., Custodio M.R., Pecoits-Filho R. (2012). Impact of cholecalciferol treatment on biomarkers of inflammation and myocardial structure in hemodialysis patients without hyperparathyroidism. J. Ren. Nutr..

[66] Bischoff-Ferrari H.A., Dawson-Hughes B., Stocklin E., Sidelnikov E., Willett W.C., Edel J.O., Stahelin H.B., Wolfram S., Jetter A., Schwager J. (2012). Oral supplementation with 25(OH)D3 versus vitamin D3: Effects on 25(OH)D levels, lower extremity function, blood pressure, and markers of innate immunity. J. Bone Miner. Res..

[67] Barker T., Martins T.B., Hill H.R., Kjeldsberg C.R., Henriksen V.T., Dixon B.M., Schneider E.D., Dern A., Weaver L.K. (2012). Different doses of supplemental vitamin D maintain interleukin-5 without altering skeletal muscle strength: A randomized, double-blind, placebo-controlled study in vitamin D sufficient adults. Nutr. Metab (Lond.).

[68] Alvarez J.A., Zughaier S.M., Law J., Hao L., Wasse H., Ziegler T.R., Tangpricha V. (2013). Effects of high-dose cholecalciferol on serum markers of inflammation and immunity in patients with early chronic kidney disease. Eur J. Clin. Nutr..

[69] Hossein-Nezhad A., Spira A., Holick M.F. (2013). Influence of vitamin d status and vitamin d3 supplementation on genome wide expression of white blood cells: A randomized double-blind clinical trial. PLoS One.

